# CAFs-Associated Genes (CAFGs) in Pancreatic Ductal Adenocarcinoma (PDAC) and Novel Therapeutic Strategy

**DOI:** 10.3390/ijms25116003

**Published:** 2024-05-30

**Authors:** Keishi Yamashita, Yusuke Kumamoto

**Affiliations:** 1 Division of Advanced Surgical Oncology, Research and Development Center for New Medical Frontiers, Kitasato University School of Medicine, Kitasato 1-15-1, Minami-ku, Sagamihara 252-0374, Japan; 2 Department of General-Pediatric-Hepatobiliary Pancreatic Surgery, Kitasato University School of Medicine, Sagamihara 252-0374, Japan; 110kumakuma@gmail.com

**Keywords:** CAFs, PDAC, SPARC, LRRC15, myCAFs, iCAFs, metabolism

## Abstract

Pancreatic ductal adenocarcinoma (PDAC) is the most aggressive cancer with striking fibrosis, and its mortality rate is ranked second across human cancers. Cancer-associated fibroblasts (CAFs) play a critical role in PDAC progression, and we reviewed the molecular understanding of PDAC CAFs and novel therapeutic potential at present. CAFs-associated genes (CAFGs) were tentatively classified into three categories by stroma specificity representing stroma/epithelia expression ratios (SE ratios). The recent classification using single cell transcriptome technology clarified that CAFs were composed of myofibroblasts (myCAFs), inflammatory CAFs (iCAFs), and other minor ones (e.g., POSTN-CAFs and antigen presenting CAFs, apCAFs). *LRRC15* is a myCAFs marker, and myCAFs depletion by diphtheria toxin induces the rapid accumulation of cytotoxic T lymphocytes (CTLs) and therefore augment PDL1 antibody treatments. This finding proposes that myCAFs may be a critical regulator of tumor immunity in terms of PDAC progression. myCAFs are located in CAFs adjacent to tumor cells, while iCAFs marked by *PDPN* and/or *COL14A1* are distant from tumor cells, where hypoxic and acidic environments being located in iCAFs putatively due to poor blood supply is consistent with *HIF1A* and *GPR68* expressions. iCAFs may be shared with SASP (secretion-associated phenotypes) in senescent CAFs. myCAFs are classically characterized by CAFGs induced by *TGFB1*, while chemoresistant CAFs with SASP may dependent on *IL6* expression and accompanied by STAT3 activation. Recently, it was found that the unique metabolism of CAFs can be targeted to prevent PDAC progression, where PDAC cells utilize glucose, whereas CAFs in turn utilize lactate, which may be epigenetically regulated, mediated by its target genes including *CXCR4*. In summary, CAFs have unique molecular characteristics, which have been rigorously clarified as novel therapeutic targets of PDAC progression.

## 1. Introduction

Pancreatic ductal adenocarcinoma (PDAC) is the most aggressive cancer with striking fibrosis, and its mortality rate is ranked second across human cancers [1]. Significant recurrent mutations were identified in *KRAS* followed by *TP53*, *CDKN2A* (*p16*), and *SMAD4* in PDAC, where mutation rates of both *KRAS* and *TP53* were confirmed as 93% and 72%, respectively [2]. The matched germline exome sequencing data clarified alterations in known germline predisposition genes such as *BRCA1* and *BRCA2*. As a result, pathogenic germline mutations were confirmed in 8% of PDAC patients in the cohort, including the most frequent germline mutations in *BRCA2*.

For the stromal landscape of *BRCA*-mutated and *BRCA*-wild-type PDAC, a comprehensive analysis was performed, revealing different cancer-associated fibroblasts (CAFs) subtypes in germline *BRCA*-mutated tumors [3]. Intriguingly, an increase in a subset of immune-regulatory clusterin-positive CAFs was detected in *BRCA*-mutated tumors. Moreover, cancer organoids and mouse models clarified that this process is mediated through the activation of heat-shock factor 1 (HSF1), the transcriptional regulator of clusterin. These findings suggest that inherited PDAC is distinct from conventional PDAC in terms of tumor microenvironment (TME) development, and target genes may be different.

Cancer metastasis is accompanied by the robust activation of the TME including CAFs [4,5]. *Secreted protein, acidic and rich in cysteine (SPARC)/Osteonectin* mRNA overexpression, was initially confirmed in colorectal cancer (CRC), and its transcripts were predominantly localized in fibroblasts adjacent to tumor cells [6]. *SPARC* overexpression has been repeatedly confirmed in diverse cancers with different histologies including hepatocellular carcinoma (HCC) [7] and esophageal squamous cell carcinoma (ESCC) [8], suggesting that *SPARC* overexpression in primary tumor tissues may be a common feature representing TME activation across human cancers.

*SPARC* overexpression in primary tumor tissues has since been demonstrated to be significantly associated with poor prognosis in melanoma [9], ESCC [8], non-small cell lung cancer (NSCLC) [10], breast cancer [11], gastric cancer (GC) [12], PDAC [13], CRC [14], and leukemia [15], and prognosis was poor in case of its stromal expression [10,13,14]. These findings together with recent single cell transcriptome analysis [16] proposed that *SPARC* relevance to poor prognosis in primary tumors may represent the stromal activation involved in cancer metastasis and prognosis.

We recently defined CAFs-associated genes (CAFGs) based on the following: (1) close association (R = 0.9 or beyond, underlined throughout this paper) with the expression of *SPARC*, a well-known stromal marker of human cancers [5,16], in cancer stroma of colorectal cancer (CRC) tumors (GSE35607); and (2) stromal specificity (stroma/epithelia expression ratio, SE ratio = 10 or beyond) like *SPARC* (SE = 17.2) [17]. CAFGs included *FAP* (SE = 20.2), *ACTA2* (SE = 20.2), and *VIM* (SE = 17.8), the well-established CAF markers [5]. These findings suggest that the stromal expression of CAFGs may represent specific subpopulations of CAFs.

The data on CRC as a human cancer representative can be exploited to other tumors because stromal components of different cancers are derived from the same individuals and postulated to be similar and shared across human body. Interestingly, such molecularly characterized CAFGs uniquely identified in CRC have also been intensively described in PDAC.

## 2. CAFGs in PDAC and Novel Therapeutic Potential

**Figure 1:**
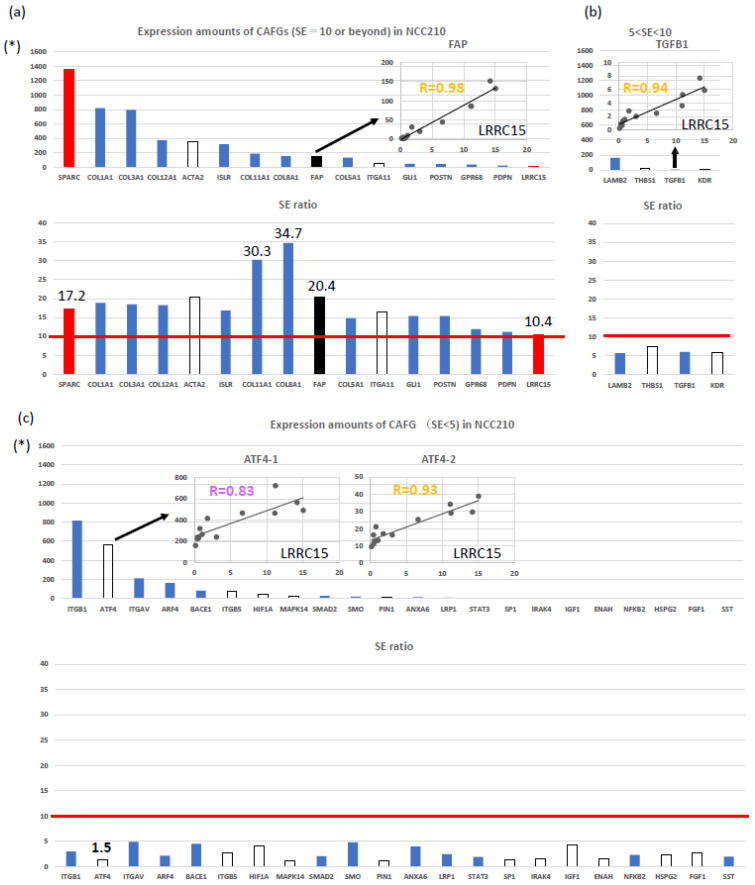
Molecular features of CAFGs, semi-CAFGs, and L-CAFGs in GSE35602. (**a**) Upper panel: Expression amounts of CAFGs (SE = 10 or beyond) in NCC210 (asterisks indicate expression amounts adjusted by GAPDHx100). Lower panel: SE ratio value is a *y*-axis. Inlet: *FAP* expression associated with *LRRC15* expression in cancer stroma (R = 0.98). Red bars represent standard genes (*SPARC* and *LRRC15*), and black bars are CAFs markers (*FAP* in the figures). (**b**) Upper panel: Expression amounts of CAFGs (5 < SE < 10) in NCC210. Lower panel: SE ratio. Inlet: *TGFB1* expression associated with *LRRC15* expression in cancer stroma (R = 0.94). (**c**) Upper panel: Expression amounts of CAFGs (SE < 5) in NCC210. Lower panel: SE ratio. Inlet: *ATF4* (probe 1 and probe 2) expression associated with *LRRC15* expression in cancer stroma (R = 0.83 and 0.93, respectively).

CAFGs with the highest expression amounts in GSE35602 were *SPARC* (Figure 1a), followed by *COL1A1* (SE = 18.9), *COL3A1* (SE = 18.5), *COL12A1* (SE = 18.3), *ACTA2*, *ISLR* (SE = 16.8), *COL11A1* (SE = 30.3), *COL8A1* (SE = 34.7), *FAP*, *COL5A1* (SE = 14.8), *ITGA11* (SE = 16.4), *GLI1 *(SE = 16.9), *POSTN* (SE = 15.4), *GPR68* (SE = 11.9), *PDPN* (SE = 11.2), and *LRRC15* (SE = 10.5), and this review article for PDAC will first summarize their clinical and functional relevance in PDAC.

**Figure 2:**
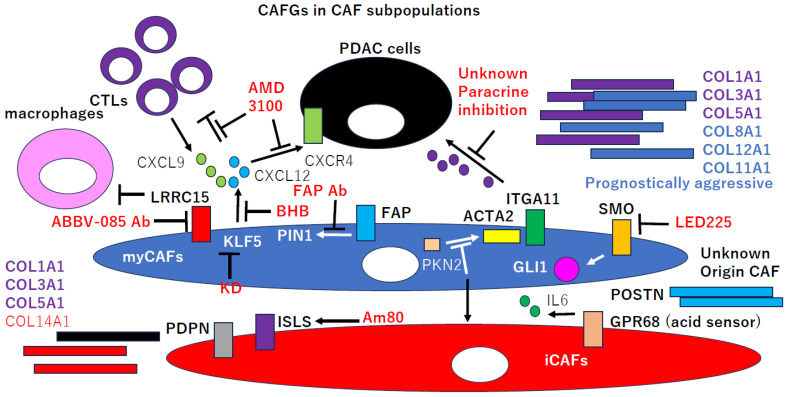
CAFGs in PDAC and novel therapeutic potential. Therapeutic potential of targets of CAFGs (*LRCC15*, *FAP*, *ACTA2*, *ITGA11*, and *GLI1*) on myCAFs and those (*PDPN*, *ISLS*, and *GPR68*) on iCAFs. CAFGs also include collagen family genes such as myCAFs collagen (*COL8A1*, *COL11A1*, and *COL12A1*), iCAF collagen (*COL14A1*), and panCAF collagen (*COL1A1*, *COL3A1*, and *COL5A1*). *POSTN* is an ECM ascribed to CAFGs with unknown origin.

Sonic Hedgehog (SHH) signaling representing *GLI1* induction was demonstrated for the first time to be uniquely activated in CAFs of PDAC tumors [18]. *Receptor Smoothened* (*SMO*, SE = 4.8) activation by SHH ligands promotes tumor growth, while SHH/SMO pathway inhibition by the SMO antagonist, LDE225, in CAFs impairs tumor growth (Figure 2) [18]. SHH inhibition reduced myofibroblasts (myCAFs) numbers and increased inflammatory CAFs (iCAF) numbers [19], which correlates with a decrease in cytotoxic T cells (CTLs) and an expansion in regulatory T cells (Tregs). These findings suggest that the SHH/SMO pathway can be a novel therapeutic target of PDAC.

*Leucine-rich repeat containing 15* (*LRRC15*) was recently identified as expressed on the stromal fibroblasts of many solid tumors including PDAC, and the LRRC15 antibody (ABBV-085 Ab) could preferentially kill cancer cells over LRRC15-positive CAFs while also increasing macrophage infiltration in the TME (Figure 2) [20]. These findings suggest that *LRRC15* may be involved in paracrine and/or juxtacrine tumor survival and the suppression of myeloid infiltration. Among the CAFGs, *LRRC15* showed the lowest expression (right red bars, Figure 1a), implying its functional relevance overriding its molecular feature.

*LRRC15*-expressed fibroblasts surround tumor islets, and its expression was induced by *TGFB1 *(SE = 6.0, Figure 1b) [21]; hence, it could be a marker of myCAFs rather than iCAFs [22,23,24]. The elevated levels of LRRC15-positive CAFs signatures correlated with poor responses to anti-PD-L1 therapy, and the depletion of LRRC15-positive CAFs markedly reduced the total tumor fibroblast content [23], which relieves the direct suppression of CTLs (Figure 2) and augments tumor regression. Collectively, these findings demonstrate that *TGFB1*-dependent LRRC15-positive CAFs dictate the tumor-fibroblast setpoint to promote tumor growth. *LRRC15* could be therefore designated as a functional CAFs maker in PDAC despite its low expression amounts.

We herein explored fCAFs-associated genes (fCAFGs) representing close association with the *LRRC15* expression (R = 0.9 or beyond) in GSE35602 according to expression amounts (140 green color gene probes, Table S1). Among the previously identified 115 *SPARC*-associated CAFGs [17], 54 genes were shared with *LRRC15*-redefined fCAFGs (Table 1). For example, *ACTA2* and *ITGA11* were removed from *IRRC15*-redefined fCAFG (blank box, Figure 1a) due to a low R index below 0.9 with *LRRC15* (R = 0.89 and 0.88, respectively), whereas a well-known PDAC CAFs marker, *FAP* [25,26,27], was closely associated with *IRRC15* (inlet figure of Figure 1a, R = 0.98).

In PDAC, FAP-positive stromal cells depleted by diphtheria toxin uncovered the antitumor effects of the antibodies of PD-L1 (CD274, SE = 2.5), where *chemokine* (*CXC motif*) *ligand 12* (*CXCL12*, SE = 1.7) explained immunosuppression by FAP-positive cells [25]. FAP was demonstrated to be a panCAFs marker but not a myCAFs marker via single cell RNA (scRNA) transcriptome analysis in mouse breast cancer [28]. Cancer cells were coated with CXCL12, while FAP-positive CAFs were the principal source of CXCL12 in the tumor. Administering AMD3100, a CXCL12 receptor, *chemokine receptor 4* (*CXCR4*, SE = 13.1) inhibitor, inhibited CXCR4 in tumor cells and moreover induced rapid T-cell accumulation (Figure 2), which acted synergistically with the PD-L1 antibody to greatly diminish PDAC tumor cells [25,26]. Nevertheless, this result did not indicate the true functional significance of *FAP* in CAFs’ contribution to cancer survival because other CAFGs expressions synchronized with the *FAP* expression were also considered to be depleted in the diphtheria toxin experiments.

Decreased ketogenesis is a signature of CRC tumor cells, and an increase in ketogenesis using a ketogenic diet (KD) decreases CXCL12 production in tumors [29]. Increasing ketogenesis by treatment with the ketone body β-hydroxybutyrate (BHB) markedly decreased the expression of *KLF5 *(SE = 0.7), which binds the *CXCL12* promoter and induces *CXCL12* expression in CAFs (Figure 2). Accordingly, KD expectedly decreased the intratumoral accumulation of immunosuppressive cells, increased infiltration of natural killer cells and CTLs, and enhanced the anticancer effects of the PD-1 blockade.

scRNA sequencing of CAFs from mouse PDAC identified subpopulations of CAFs with a decreased expression of *CXCL12* and an increased expression of the T cell-attracting chemokine, *CXCL9*, in association with T-cell infiltration (Figure 2). TNFA (SE = 2.0) and IFNG (SE = 2.4) containing conditioned media from activated CTLs converted stromal fibroblasts from a CXCL12+/CXCL9− immune-suppressive phenotype into a CXCL12−/CXCL9+ immune-activating phenotype, where recombinant IFNG and TNFA acted together to augment *CXCL9* expression, whereas TNFA alone suppressed *CXCL12* expression [30].

In PDAC, *COL1A1*, *COL3A1*, and *COL5A1* were expressed in all CAF subtypes (panCAFs), whereas *COL11A1*, *COL12A1*, and *COL8A1* were specific to myCAFs overexpressed adjacent to tumor cells (Figure 2, Table 2) [31]. Interestingly, our recent prognostic analysis using GSE17538 clarified that COL family genes representing myCAFs collagen markers were more aggressive as compared to panCAFs collagen markers in colon cancer [17], suggesting that myCAFs collagens may play a more important role than panCAFs collagens in cancer aggressiveness.

Integrin (ITG) family genes are receptors for cognitive ligands, such as COL family genes and other ECM components like *periostin* (*POSTN*). *POSTN* was included in CAFGs (Figure 1a), however *POSTN*-positive CAFs were not correlated with the published myCAFs/iCAFs classification in PDAC [32] (Table 2). This finding suggests that the minor CAFs subpopulation may be involved in TME uniqueness. Among integrin (ITG) family genes, *ITGA11* alone exhibited the most highly stroma-specific feature (SE = 16.4) (Figure 1a) as compared to other ITG family genes such as *ITGB1* (SE = 3.0), *ITGAV* (SE = 4.8), and *ITGB5* (SE = 2.7) (Figure 1c), although *ITGB1* and *ITGAV* had much more abundant expression amounts than *ITGA11* in cancer stroma. These findings suggest that *ITGA11* may play a unique role in the stromal biology in CAFs.

Interestingly, ITGA11 is immunolocalized to ACTA2-positive CAFs, hence putatively representing myCAFs [22,33]. This finding is consistent with the fact that *TGFB1* robustly induced *ITGA11* expression. However, conditioned mediums (CMs) from shITGA11 pancreatic satellite cells (PSCs) caused tumor cells to migrate and invade less than their counterpart, indicating the paracrine effects of this myCAFs marker, *ITGA11*, to cancer invasion by unknown secretion factors (Figure 2).

*Rho effector protein kinase N2* (*PKN2*) is critical for pancreatic satellite cells’ (PSCs) myCAFs differentiation. The loss of *PKN2* is associated with reduced PSCs proliferation, contractility, and ACTA2 stress fibers (Figure 2) [34]. In spheroid co-cultures with PDAC cells, the loss of *PKN2* prevents PSC invasion but, counter-intuitively, promotes invasive cancer cell outgrowth. *PKN2* deletion induces a myCAFs to iCAFs switch in the PSC matrisome signature (Figure 2). Furthermore, the deletion of *PKN2* in the pancreatic stroma induces more locally invasive tumors, and *PKN2* knockout (KO) matrisome signature predicts poor outcomes in PDAC. This finding suggests that iCAFs molecular signatures may represent a poor prognosis in PDAC.

Among the COL family genes, *COL14A1* (SE = 11.0) expression was specific to iCAFs (Figure 2, Table 2) [31], however *COL14A1* expression was not strongly associated with CAFGs and differently from other collagens. On the other hand, Podoplanin (PDPN)-positive CAFs among CAFGs identified an iCAF-like subset in PDAC, and the combination of PDPN and POSTN was associated with specific TME features in terms of stromal abundance and immune cell infiltrates (Table 2) [32]. Recently, such minor CAFs subtypes from a defined cell of origin played unique roles in establishing TME in PDAC [35].

A new population of CAFs that express MHC class II and CD74 was discovered, which was designated as “antigen-presenting CAFs, apCAFs”, and they activate CD4-positive T cells and are derived from mesothelial cells [36]. During PDAC progression, mesothelial cells form apCAFs by downregulating mesothelial features and gaining fibroblastic features—a process induced by IL1 and TGFB1. apCAFs directly ligate and induce naive CD4-positive T cells into Tregs. Moreover, treatment with an antibody targeting mesothelin can effectively inhibit mesothelial cells from apCAFs transition and Treg formation induced by apCAFs in PDAC [37] (Table 2).

CAFs have increased expressions of *GPR68* (a proton-sensing GPCR, G-protein coupled receptor) in PDAC tumors. The co-culture of PSCs with PDAC cells, or incubation with TNFA, induced *GPR68* expression. GPR68 activation (by decreasing the extracellular pH representing microenvironments with poor blood supply) enhanced *IL6* (SE = 10.0) expression (Figure 2) [38,39]. These findings suggest that GPR68 may be a subpopulation of iCAFs (Figure 2).

Differently from many CAFGs, *Meflin* (*ISLS*) is a marker of repressive CAFs that suppress PDAC progression [40]. A chemical library screen identified Am80 as a reagent that effectively induced *ISLS* expression in CAFs [41] (Figure 2). Am80 administration improved the sensitivity of PDAC to chemotherapeutics accompanied by increases in tumor vessel area and intratumoral drug delivery. Mechanistically, *ISLS* was involved in the suppression of tissue stiffening by interacting with lysyl oxidase (LOX) to inhibit its collagen crosslinking activity.

## 3. CAFGs Collagens in PDAC and Novel Therapeutic Potential

**Figure 3:**
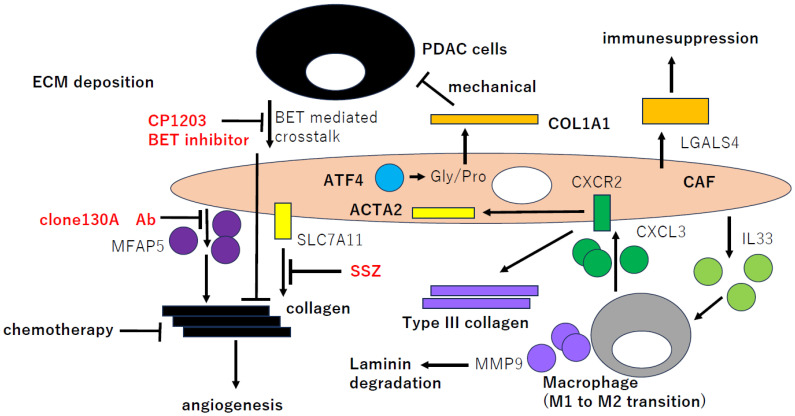
CAFGs collagens in PDAC and novel therapeutic potential. Therapeutic potential of targets of collagen production and deposits by MFAP5, BET, and SLC11A7. CAFGs (*LRCC15*, *FAP*, *ACTA2*, *ITGA11*, and *GLI1*) in myCAFs and those (*PDPN*, *ISLS*, and *GPR68*) in iCAFs. *COL1A1* is produced by *ATF4*, and Type III collagen is produced and mediated uniquely by CXC3/CXCR2 in PDAC. *LGASL4* is an ECM involved in immune suppression in PDAC.

The expression of type I, III, IV, and V collagen was reduced in PDAC tissues after effective neoadjuvant chemotherapy (NAC) (Figure 3), suggesting that collagen deposition may play a critical role in chemotherapeutic sensitivity. The bioinformatics approach provided comprehensive insights into NAC-induced matrix remodeling, which showed Ephrin-A signaling as a likely pathway and Ephrin-A5 (encoded by EFNA5, SE = 1.9) as a crucial ligand. Interestingly, effective NAC reduced the number of Ephrin-A5 positive cells, which inversely correlated with tumor shrinkage [42].

Collagen synthesis and deposition may be predominantly regulated in CAFs in PDAC by various molecular mechanisms (Figure 3). For example, microfibril-associated protein 5 (MFAP5, SE = 11.3) is secreted predominately by CAFs, and the MFAP5 blockade inhibits fibrosis and enhances chemosensitivity in PDAC [43]. The BET (bromodomain and extraterminal) family inhibitor, CP1203, also induced the expression of ECM genes (matrisome) including *COL1A1* [44]. Alternatively, the specific expression of *SLC7A11* (SE = 1.5) in cancer stroma tumors is independently prognostic of poorer overall survival, and PDAC-derived CAFs are highly dependent on *SLC7A11*. SLC7A11 inhibition by sulfasalazine (SSZ) significantly decreases CAFs proliferation, reduces their resistance to oxidative stress, and inhibits their ability to remodel collagen (Figure 3) and support PDAC cell growth [45].

ATF4 (SE = 1.5) is a major effector of the integrated stress response. Single-cell transcriptomics of tumors grown in ATF4 knockout (KO) mice uncovered a reduction in activation markers in perivascular CAFs. ATF4 KO fibroblasts displayed significant defects in collagen synthesis and deposition and a reduced ability to support angiogenesis. Mechanistically, *ATF4* also regulates *COL1A1* expression and levels of glycine and proline, which are the major amino acids in collagen (Figure 3) [46]. Intriguingly, *ATF4* expression was abundant (Figure 1c), and the best probe showed close association (R = 0.93) with myCAFs marker, *LRRC15*, expression in GSE35602 (inlet of Figure 1c).

*CXCL3* (SE = 0.64) was highly upregulated in *IL-33* (SE = 0.84)-stimulated macrophages that were the primary source of *CXCL3* (Figure 3), and *CXCL3* was correlated with poor survival in PDAC [47]. The highest level of *CXCL3* was found in PDAC relative to other cancer types, and its receptor *CXCR2* (*IL8RB*, SE = 1.8) was almost exclusively expressed in CAFs. The activation of CXCR2 by *CXCL3* induced a CAFs-to-myoCAFs transition, and *ACTA2* as well as type III collagen were uniquely upregulated by the CXCL3-CXCR2 signaling for myoCAF-driven PDAC metastasis. Moreover, *MMP9* (SE = 10.8) expressed in IL33-stimulated macrophages degrades the laminin of the basement membrane to promote metastasis [48].

## 4. Semi-CAFGs in PDAC and Novel Therapeutic Potential

*LAMB2* (*Laminin5*) (SE = 5.7), *TGFB1*, *THBS1*, and *KDR* (VEGF receptor, SE = 5.9) expressions were also closely (R = 0.9 or beyond) associated with a stromal marker, *SPARC*, and/or *LRRC15* expressions in the cancer stroma of GSE35602 (Figure 1b); however, they were not defined as CAFGs due to the SE ratio being below 10 [17] (Figure 1b). The four genes however could be designated as semi-CAFGs because they exhibited stroma-prone expression features (SE ratio = 5 or beyond).

**Figure 4:**
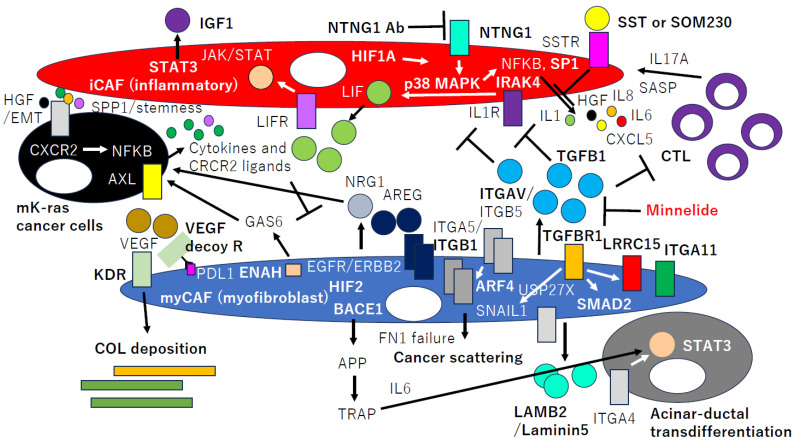
Semi-CAFGs and L-CAFGs in PDAC and their novel therapeutic potential. *TGFB1* is involved in myCAFs activation and iCAFs suppression and is suppressed by Minnelide. *TGFB1* induces other CAFGs such as *LRRC15* and *ITGA11* as well as *SNAIL1*. For CAFs activation, ITGA5/ITGB1 internalization by *ARF4* is essential and mediated by ITGAV/ITGB5. CAFs-secreted LAMB2 (Laminn5) may be critical for acinar-ductal transdifferentiation and STAT3 activation and is mediated by ITGA4. *KDR* (VEGFR) could be targeted for collagen deposition control. HIF1A is critical for iCAFs activation, while HIF2 is important for myCAFs activation. myCAFs subpopulations utilize EGFR/ERBB2 activation by *AREG* and/or *NRG1*, while iCAFs use p38 MAPK (MAPK14)/NFKB activation pathway to induce SASP fibroblast phenotypes.

Proteomic and transcriptomic data integration revealed a LAMB2 (Laminin5)/ITGA4/STAT3 axis in the pancreatic acinar cells responsible for CAFs-derived LAMB2-mediated acinar-to-ductal cell trans-differentiation (Figure 4). This finding proposes that LAMB2-positive CAFs may play an important role in the initial carcinogenesis of PDAC [49].

It has been demonstrated that TGFB1 induces many myCAFs markers, such as *LRRC15* and *ITGA11*, as described earlier. It also requires the production of numerous desmoplasia-ECM (D-ECM), but it is unexpectedly dispensable for D-ECM-induced CAF activation [50], which depends on the ITGAV (SE = 4.9)/ITGB5 (SE = 2.7) redistribution of ITGA5 (SE = 6.4)/ITGB1 (SE = 3.0) inside fibroblasts (Figure 4). Interestingly, stromal localization and the levels of active SMAD2 (SE = 2.1) and ITGA5/ITGB1 distinguish patient-protective from patient-detrimental desmoplasia and foretell tumor recurrences.

Fibronectin (FN1, SE = 2.0) assembled by CAFs mediates CAFs-cancer cells association and directional migration. CAFs produce an FN1-rich ECM with anisotropic fiber orientation, which guides cancer cells to migrate directionally. CAFs align the FN1 matrix by increasing platelet-derived growth factor receptor α (PDGFA, SE = 2.1)-mediated contractility and traction forces, which are transduced to FN1 through ITGA5/ITGB1. Cancer cells use ITGAV to migrate efficiently and directionally on CAF-derived matrices [51]. Hence, ITGA5/ITGB1 internalization may result in cancer cell random scattering due to anisotropic loss.

Minnelide revealed the suppression of the TGFB1 signaling pathway in CAFs (Figure 4), resulting in an apparent reversal of their activated state to a quiescent, non-proliferative state [52]. Tumor epithelial cells (TEC) exposed to media conditioned by Minnelide-treated CAFs exhibited a decrease in oncogenic signaling, as manifested by the downregulation of the transcription factor SP1 in TEC. This inhibition was rescued by treating TEC with TGFB1, suggesting that Minnelide may be promising for the regulation of CAFs.

CAFs subtypes are well represented by either myCAFs or iCAFs, and TGFB1 and IL1 promote such CAFs’ heterogeneity [53]. IL1 induces the *leukemia inducible factor* (*LIF*, SE = 1.8) expression and downstream JAK/STAT activation to generate iCAFs and demonstrated that TGFB1 antagonizes this process by downregulating IL1R1 (SE = 6.9) expression and promoting differentiation in myCAF from iCAF (Figure 4).

SNAIL1 (SNAI1, SE = 2.8) has been considered to be a relevant transcriptional factor required for the activation of CAFs. The SNAIL1 stability was upregulated by TGFB1 during the epithelial mesenchymal transition (EMT), and USP27X, a deubiquitinase of SNAIL1, was required for the TGFB1-induced expression of SNAIL1 in CAFs [54]. Moreover, CAFs exposed to gemcitabine significantly increase the release of extracellular vesicles (EVs) called exosomes, which increase SNAIL1 and promote proliferation and drug resistance [55]. Nevertheless, *SNAIL1* expression was not so strongly associated with CAFGs expressions (R < 0.9).

Epidermal growth factor receptor/Erb-B2 receptor (EGFR, SE = 1.1/ERBB2, SE = 1.4) signaling is induced by TGFB1 in myCAFs through an autocrine process mediated by amphiregulin (AREG, SE = 0.61) (Figure 4). The inhibition of this network in PDAC differentially impacts distinct CAFs subtypes, providing insights into mechanisms underpinning their heterogeneity. Remarkably, EGFR-activated myCAFs promote PDAC metastasis [56]. On the other hand, the CAFs-derived NRG1 (SE = 3.6) activation of cancer cell ERBB2 and ERBB3 receptor tyrosine kinases as a paracrine mechanism supports mutant KRAS-independent growth (Figure 4) [57]. *AREG* and *NRG1* expressions in cancer stroma were not closely associated with CAFGs (*LRRC15*, R = 0.04 and 0.03, respectively) at all, suggesting that EGFR ligands-expressed CAFs may be unique subpopulations among CAFs.

Formation of the Annexin A6/LDL receptor-related protein 1/thrombospondin 1 (ANXA6, SE = 3.9/LRP1, SE = 2.5/*THBS1*, SE = 6.8) complex was restricted to CAFs and required physio-pathologic culture conditions that improved tumor cell survival and migration [58]. Increased PDAC aggressiveness was dependent on the tumor cell-mediated uptake of CAF-derived ANXA6-positive EVs carrying the ANXA6/LRP1/*THBS1* complex. The depletion of ANXA6 in CAFs impaired complex formation and subsequently impaired PDAC metastasis, while the injection of CAFs-derived ANXA6-positive EVs enhanced tumorigenesis.

Patients with poor prognoses also have high *PIGF*/*VEGF* (SE = 1.2) expression and an increased number of PIGF/VEGF receptor FLT1 (SE = 4.4)/KDR-expressing CAFs, associated with enhanced collagen deposition [59]. For the VEGF receptor, *KDR*, but not *FLT1*, was included among semi-CAFGs with SE ratios between 5 and 10 (Figure 1b). Based on these findings, the VEGF decoy receptor (Ate-Grab) was made by fusing atezolizumab (anti-PD-L1) to VEGF-Grab to target PD-L1 (SE = 2.5)-expressing CAFs, which exerted anti-tumor and anti-fibrotic effects on PDAC models via the PD-L1-directed PlGF/VEGF blockade (Figure 4).

Recently, the expression of CD105 (ENG, endoglin, SE = 6.0) demarks two functionally distinct pancreatic fibroblast lineages. Whereas CD105-positive PDAC CAFs are permissive for tumor growth in vivo, CD105-negative PDAC CAFs are highly tumor suppressive [60]. CD105 was not herein defined as CAFGs and/or fCAFGs because its expression was not so strongly associated with *LRRC15* expression in GSE35602 (R = 0.76).

## 5. CAFGs with Low SE (L-CAFGs) and Novel Therapeutic Potential in PDAC

Although the SE ratio was low (below 5), many genes critical for CAFs activation in PDAC were closely (R = 0.9 or beyond) associated in expression with *SPARC* and/or *LRRC15* in GSE35602 (Figure 1c, Table S1), and they could be designated as CAFGs with low SE (L-CAFGs) in this paper. The L-CAFGs according to expression amounts are shown in Figure 1c, ranked as top L-CAFGs of *ITGB1* followed by *ATF4*, *ITGAV*, *ARF4*, *BACE1*, *ITGB5*, *HIF1A*, *MAPK14 (p38)*, *SMAD2*, *SMO*, *PIN1*, *ANXA6*, *LRP1*, *STAT3*, *SP1*, *IRAK4*, *IGF1*, *ENAH*, *NFKB2*, *HSPG2*, *FGF1*, and *SST*. Among them, we will shortly summarize the following genes in order of expression amounts, including *ARF4*, *BACE1*, *HIF1A*, *p38*, *PIN1*, *STAT3*, *SP1*, *IRAK4*, *IGF1*, *ENAH*, *NFKB2*, *HSPG2*, *FGF1*, and *SST*, because they have not been described yet in this paper.

Ligand-engaged ITGA5/ITGB1 are internalized under the control of the Arf subfamily GTPase, ARF4 (SE = 2.2), and are trafficked to nearby late endosomes/lysosomes [61] (Figure 4). Nutrient depletion within tumor tissues is considered to promote the subnuclear accumulation and endocytosis of ligand-engaged ITGA5/ITGB1 via inhibition of mTORC1. This regulatory interaction between mTORC1 and integrin trafficking in combination and in invasive cell migration indicate interesting links between nutrient signaling and metastasis.

CAFs induce neutrophil extracellular trap (NET) formation within tumors. These tumor-induced NETs (t-NETs) are driven by a ROS-mediated pathway dependent on CAFs-derived Amyloid β (APP, SE = 1.5), which is a peptide implicated in inflammatory disorders. The inhibition of NETosis in murine tumors skews neutrophils to an anti-tumor phenotype, preventing tumor growth. CAFs juxtaposed to NETs in PDAC show elevated *BACE1* (*memapsin2*, *Secretase β*) (SE = 4.5), which correlates with poor prognoses [62] (Figure 4). Neutrophil-mediated fibroblast-tumor cell IL6/STAT3 signaling underlies the association between neutrophil-to-lymphocyte ratio dynamics (Figure 4) [63].

A dual recombinase mouse model to delete HIF1A (SE = 4.1) or HIF2A (EPAS1, SE = 2.6) was made in ACTA2-expressing CAFs (myCAFs) arising within spontaneous PDAC tumors. The CAFs-specific deletion of HIF2A, but not HIF1A, suppressed PDAC tumor progression and growth. The deletion of CAFs-HIF2 modestly reduced tumor fibrosis and significantly decreased the intratumoral recruitment of immunosuppressive M2 macrophages and Tregs. Treatment with the clinical HIF2 inhibitor PT2399 also significantly reduced macrophage chemotaxis and M2 polarization and improved tumor responses to immunotherapy in syngeneic PDAC mouse models [64].

iCAFs displayed a hypoxic gene expression and biochemical profile and were enriched in hypoxic regions of PDAC tumors, while myCAFs were excluded from these regions [22]. Hypoxia led fibroblasts to acquire an inflammatory gene expression signature and synergized with cancer cell-derived cytokines to promote an iCAFs phenotype in a HIF1A-dependent fashion. HIF1A stabilization was sufficient to induce an iCAFs phenotype in stromal cells introduced into PDAC organoid co-cultures and to promote PDAC tumor growth (Figure 4) [65]. These findings indicate that hypoxia-induced HIF1A is a regulator of CAFs’ heterogeneity via the induction of iCAFs and promotion of tumor progression in PDAC.

Chemoresistant immortalized CAFs (R-CAFs) were generated by continuous incubation in gemcitabine, and R-CAFs had increased expressions of various inflammatory mediators similar to the previously described senescence-associated secretory phenotype (SASP). SASP mediators were found to be upregulated in response to short duration treatment with gemcitabine in CAFs, and such CAFs may be similar to iCAFs. The inhibition of stress-associated MAPK signaling (p38 MAPK, SE = 1.2) attenuated SASP in iCAF [66] (Figure 4).

NetG1 (NTNG1, SE = 1.8)-positive CAFs support PDAC survival through a NetG1-mediated effect on glutamate/glutamine metabolism [67]. Also, NetG1-positive CAFs are intrinsically immunosuppressive and inhibit the natural killer cell-mediated killing of tumor cells. These protumor functions are controlled by a signaling circuit downstream of NetG1, which is comprised of AKT/4E-BP1 and p38 MAPK/FRA1. Finally, blocking NetG1 with a neutralizing antibody stunts in vivo tumorigenesis. These findings suggest that NTNG1 positive CAF may be iCAF (Figure 4).

The prolyl isomerase PIN1 (SE = 1.1), whose overexpression in CAFs has not been fully profiled yet, plays critical roles in tumor initiation and progression. A DNA-barcoded micellular system (DMS) functionalized with CAFs-targeting anti-FAP antibodies (antiCAFs-DMS) can selectively inhibit PIN1 in CAFs (Figure 2), leading to efficacious but transient tumor growth inhibition [27].

PDAC cells can induce DNA methylation in CAFs [68]. The *SOCS1* promoter’s DNA methylation and downregulation in CAFs activated STAT3 (SE = 2.2) and induced *insulin-like growth factor-1* (*IGF1*, SE = 7.4) expression to support PDAC cell growth. Moreover, CAFs facilitated methylation-dependent growth of PDAC tumor xenografts in mice. The ability of patient-derived CAFs with *SOCS1* methylation to promote PDAC growth was more robust than that of CAFs without *SOCS1* methylation.

Inhibition of the IL1 receptor-associated kinase 4 (IRAK4, SE = 1.6) suppresses NFKB activity and promotes responses to chemotherapy in PDAC cells. CAFs in PDAC tumors robustly express activated IRAK4 and NFKB. The *IRAK4* expression in CAFs promoted NFKB activity, drove tumor fibrosis, and supported PDAC cell proliferation, survival, and chemoresistance [69]. The cytokine array analysis of CAFs and microarray analysis of PDAC cells identified IL1B (SE = 5.7) as a key cytokine that activated IRAK4 in CAFs. Targeting IRAK4 or IL1B rendered PDAC tumors less fibrotic and more sensitive to gemcitabine.

hMENA (ENAH, SE = 1.7) is a member of the actin regulatory protein of the Ena/VASP family, and a LC-MS/MS proteomic analysis revealed that CAFs that overexpress ENAHΔv6 secrete the AXL ligand GAS6 (SE = 0.94), favoring the invasiveness of PDAC cells [70]. *ENAH*/GAS6/AXL gene expression signature is associated with a poor prognosis in PDAC.

*Perlecan* (*HSPG2*, SE = 2.3) was identified as a key component of pro-metastatic environments and derived from CAFs. Depleting *perlecan* in the stroma combined with chemotherapy prolongs mouse survival, supporting it as a potential target for anti-stromal therapies in PDAC [71].

The *acidic fibroblast growth factor* (*FGF1*, SE = 2.8) derived from CAF cooperates with cancer cell-autonomous signals to increase MYC levels and promoter occupancy and activity. *FGF1* is necessary and sufficient for the paracrine regulation of MYC protein stability, signaling through AKT and GSK-3β to increase the MYC half-life. Patient specimens reveal a strong correlation between stromal CAFs’ content and MYC protein levels in the neoplastic compartment and identify CAFs as the specific source of FGF1 in the tumor microenvironment [72].

CAFs selectively express SST (SE = 2.0). The SOM230 analogue (Pasireotide) activates the SST receptor (Figure 4) and inhibits the mTOR/4E-BP1 pathway and the resultant synthesis of secreted proteins including IL-6. Consequently, tumor growth and chemoresistance in nude mice xenografted with PDAC cells and CAFs, or with pieces of resected human PDAC, are reduced when chemotherapy (gemcitabine) is combined with SOM230 treatment. While gemcitabine alone has marginal effects, SOM230 is permissive to gemcitabine-induced cancer cell apoptosis and acts as an antifibrotic agent [73].

## 6. CAFs’ Metabolism in PDAC and Novel Therapeutic Potential

**Figure 5:**
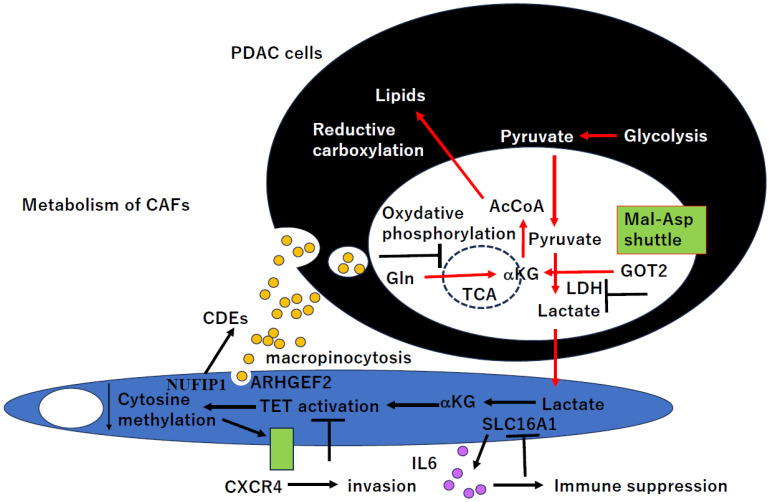
CAFs metabolism in PDAC and novel therapeutic potential. PDAC cells suppressed oxidative phosphorylation (TCA cycle), while glycolysis to generate lactate and reductive carboxylation are uniquely activated and mediated by CAFs-derived exosomes (CDEs) in PDAC cells. Lactate from PDAC cells were utilized by CAFs, and *CXCR4* expression is epigenetically affected by this unique metabolism of CAFs.

CAFs’ metabolism is unique, and the unique features could be targeted as a novel therapeutic strategy in PDAC. CDEs inhibit mitochondrial oxidative phosphorylation (TCA cycle) in cancer cells (Figure 5), thereby increasing glycolysis and glutamine (Gln)-dependent reductive carboxylation [74]. CDEs contain intact metabolites, including amino acids, lipids, and TCA-cycle intermediates that are avidly utilized by cancer cells for central carbon metabolism.

The widespread loss of cytosine methylation was associated with the overexpression of various inflammatory transcripts including CXCR4 [75]. A co-culture of neoplastic cells with CAFs led to increased invasiveness that was abrogated by the inhibition of *CXCR4*. Lactate produced by neoplastic cells leads to the increased production of alpha-ketoglutarate (αKG), and αKG in turn mediated the activation of the demethylase TET enzyme and led to decreased cytosine methylation and increased hydroxymethylation for *CXCR4* overexpression (Figure 5). Intriguingly, TET-deficient MSCs also inhibited tumor growth in vivo.

Cytosine methylation of the promoter CpG islands of the individual genes is involved in gene silencing, which is mediated through *DNMT1* and *DNMT3A* in human cancers [76]. In CAFs, on the other hand, hypomethylation for CpG shore, but not CpG islands, was associated with *CXCR4* overexpression, and *CXCR4* knockdown suppressed the cancer cell invasion of PDAC cells (Panc1) [75]. Moreover, hypomethylation of *CXCR4* by demethylase TET is accompanied by increased hydoxymehylation (5hmC) during de novo differentiation of mesenchymal stem cells (MSCs) to CAFs because 5hmC is considered to be intermediate to hypomethylation and/or itself may be required for transcription regulation [77].

When PDAC cells are exposed to a nutrient-depleted TME, they can acquire nutrients via macropinocytosis, which is an endocytic form of protein scavenging that functions to support cancer metabolism. Macropinocytosis is also operational in the PDAC tumor stroma. Glutamine (Gln) deficiency triggers the macropinocytic uptake in PDAC CAFs (Figure 5), and stromal macropinocytosis is potentiated via the enhancement of cytosolic Ca^2+^ and dependent on ARHGEF2 (SE = 2.2) and CaMKK2-AMPK (PRKAA1, SE = 1.3) signaling [78].

Glutamate (Glu)-oxaloacetate transaminase 2 (GOT2, SE = 0.92) is part of the malate-aspartate shuttle, a mechanism by which cells transfer reducing equivalents from the cytosol to the mitochondria. GOT2 is a key component of the mutant KRAS (KRAS*)-mediated rewiring of glutamine metabolism in PDAC (Figure 5). CAFs release pyruvate, and culturing *GOT*2 KD cells in CAFs conditioned media (CM) rescued proliferation in vitro [79]. Blocking pyruvate import or pyruvate-to-lactate reduction prevented the rescue of *GOT2* KD in vitro by exogenous pyruvate or CAFs CM.

CAFs are critical for survival from PDAC on glutamine deprivation, in which a role for nucleosides is secreted by CAFs through autophagy in a nuclear fragile X mental retardation-interacting protein 1 (NUFIP1, SE = 1.5)-dependent manner (Figure 5), increasing glucose utilization and promoting the growth of PDAC [80]. Moreover, CAFs-derived nucleosides induced glucose consumption under glutamine-deprived conditions and displayed a dependence on MYC.

LDHA (SE = 1.1) depletion suppressed tumor growth in a CAFs-rich murine PDAC model. A coculture of CAFs with PDAC cells revealed that most of the glucose was taken up by the tumor cells and that CAFs consumed lactate via the monocarboxylate transporter 1 (SLC16A1, SE = 2.8) to enhance proliferation through the TCA cycle. Moreover, lactate-stimulated CAFs upregulated IL-6 expression and suppressed cytotoxic immune cell activity synergistically with lactate [81].

## 7. Conclusions

In this review article, we classified CAFGs into three categories including (conventional) CAFGs, semi-CAFGs, L-CAFGs and described their unique molecular metabolism in PDAC. Such unique CAFGs and metabolites could be novel molecular targets against tumor stroma instead of tumor cells themselves to inhibit cancer progression, putatively through the suppression of tumor cells and/or augmentation of tumor immunity. Hence, a diverse range of suppression points have been clarified in PDAC, while the most critical points have remained elusive.

In this review article, there are several limitations. We used molecular characteristics of CAFGs inferred from CRC tumors (GSE35602), which includes desmoplastic and non-desmoplastic ECMs, while PDAC tumors may more frequently include desmoplastic ECMs compared to CRC tumors. However, invasive cancer tumors are not appropriate for a microdissection analysis due to potential contaminations of tumor cells in TME, especially for PDAC. Nevertheless, intriguingly, many CAFGs identified in GSE35602 have been described in PDAC in this review. Nevertheless, this article summarized the current understanding of PDAC CAFs for their novel therapeutic strategy, and in the near future, more accurate clinical relevance and further functional validation may identify the best therapeutic targets for PDAC.

## Figures and Tables

**Table 1:** fCAFG molecular features identified in GSE35602 (beyond 40 in NCC210).

Expression Order	SYMBOL	NCC210	R Index with fCAFG (LRCC15)	SE Ratio	Figure 1a
Order of expression amounts	LRRC15	14	1.00	10.5	Yes
26	CMTM3	179	0.99	13.6	
25	LOXL1	182	0.98	18.3	
34	FAP	152	0.98	20.4	Yes
10	COL3A1	797	0.98	18.5	Yes
5	COL1A2	942	0.98	21.9	
47	MXRA5	57	0.98	14.1	
2	SPARC	1356	0.97	17.2	Yes
30	TAGLN	162	0.97	19.6	
21	INHBA	280	0.97	20.9	
43	COL8A2	65	0.97	21.2	
49	C1QTNF5	48	0.97	14.0	
9	COL1A1	819	0.96	18.9	Yes
11	CTSK	682	0.96	17.2	
52	POSTN	44	0.96	15.4	Yes
27	RAB34	174	0.95	10.7	
8	C1S	853	0.95	21.4	
20	AEBP1	284	0.95	23.1	
45	SSPN	60	0.95	12.4	
12	COL5A2	674	0.95	18.4	
1	IGFBP7	1443	0.95	11.6	
51	GLI1	47	0.95	16.9	Yes
32	AL359062 (COL8A1)	153	0.95	34.7	Yes
4	FBLN1	1004	0.95	30.4	
41	CFHR1	75	0.94	17.6	
48	AK022110 (GPX8)	56	0.94	15.9	
17	ISLR	318	0.94	16.8	Yes
42	FBLN2	65	0.94	17.7	
16	DKK3	365	0.94	13.9	
33	SERPINF1	152	0.94	11.7	
3	SPON2	1194	0.94	12.0	
19	PMP22	287	0.93	12.4	
36	COL5A1	132	0.93	14.8	Yes
50	COX7A1	48	0.93	15.7	
22	ANTXR1	247	0.93	24.3	
39	CR603437 (GNB4)	79	0.93	12.9	
13	MXRA8	460	0.93	19.3	
18	COL6A3	298	0.93	16.2	
14	NNMT	442	0.93	20.6	
37	C16orf30 (TMEM204)	130	0.93	14.3	
40	TSPAN4	78	0.93	13.0	
15	COL12A1	374	0.93	18.3	Yes
35	PRKCDBP (CAVIN3)	137	0.92	12.6	
23	C1R	243	0.92	17.8	
28	C10orf10 (DEPP1)	169	0.92	14.3	
29	MYLK	162	0.92	17.2	
24	COL11A1	188	0.92	30.3	Yes
6	THBS2	893	0.91	30.3	
38	HOPX	104	0.91	17.5	
31	RAB31	159	0.91	16.8	
46	BHLHB3	58	0.91	10.6	
53	ITIH5	44	0.91	12.3	
54	PLXND1	43	0.90	10.8	
7	LUM	879	0.90	18.3	
44	MEG3	61	0.90	22.2	

**Table 2:** Subtypes of CAFs, their molecular markers, functions, and their roles in PDAC.

	Molecular Markers	Function	Roles in PDAC Progression
myCAFs	ACTA2, COL8A1, COL11A1	TGFB1 pathway activation	immune suppression
iCAFs	COL14A1, PDPN	SASP (senescence-associated) secretion phenotypes, chemoresistance	hypoxia response, immune mobilization
POSTN CAFs	POSTN	macrophage interaction	tumor cell aggressiveness
apCAFs	MHC class II, CD74	Treg mobilization	immune suppression

## Supplementary Materials

The following supporting information can be downloaded at: https://www.mdpi.com/article/10.3390/ijms25116003/s1.
